# Aliskiren Improved the Endothelial Repair Capacity of Endothelial Progenitor Cells from Patients with Hypertension via the Tie2/PI3k/Akt/eNOS Signalling Pathway

**DOI:** 10.1155/2020/6534512

**Published:** 2020-05-28

**Authors:** Shun Yao, Chen Su, Shao-Hong Wu, Da-Jun Hu, Xing Liu

**Affiliations:** ^1^Department of Hypertension and Vascular Disease, The First Affiliated Hospital, Sun Yat-Sen University, Guangzhou, Guangdong Province 510080, China; ^2^Guangdong Cardiovascular Institute, Guangdong Provincial Peolple's Hospital, Guangdong Academy of Medical Science, Guangzhou, Guangdong Province 510080, China; ^3^Department of Ultrasonography, The First Affiliated Hospital, Sun Yat-Sen University, Guangzhou, Guangdong Province 510080, China; ^4^Department of Cardiology, The Affiliated Chenzhou Hospital (The First People's Hospital of Chenzhou), Nanhua University, Chenzhou, Hunan Province 423000, China; ^5^Department of Cardiovascular, The Third Affiliated Hospital, Sun Yat-Sen University, 600 TianHe Road, Guangzhou 510000, China

## Abstract

**Background:**

Studies show that aliskiren exerts favourable effects not only on endothelial progenitor cells (EPCs) but also on endothelial function. However, the mechanism of the favourable effect of aliskiren on EPCs from patients with hypertension is unclear and remains to be further studied.

**Methods:**

The object of this study was to investigate and assess the in vitro function of EPCs pretreated with aliskiren. After treated with aliskiren, the human EPCs were transplanted into a nude mouse model of carotid artery injury, and the in vivo reendothelialization of injured artery was estimated by staining denuded areas with Evans blue dye via tail vein injection.

**Results:**

We found that aliskiren increased the in vitro migration, proliferation, and adhesion of EPCs from patients with hypertension in a dose-dependent manner and improved the reendothelialization capability of these EPCs. Furthermore, aliskiren increased the phosphorylation of Tie2, Akt, and eNOS. After the blockade of the Tie2 signalling pathway, the favourable effects of aliskiren on the in vitro function and in vivo reendothelialization capability of EPCs were suppressed.

**Conclusions:**

This study demonstrates that aliskiren can improve the in vitro function and in vivo reendothelialization capability of EPCs from patients with hypertension via the activation of the Tie2/PI3k/Akt/eNOS signalling pathway. These findings further indicate that aliskiren is an effective pharmacological treatment for cell-based repair in hypertension-related vascular injury.

## 1. Introduction

As a major cardiovascular disease, hypertension usually impairs target organs and increases the risk of cardiovascular events. Endothelial dysfunction and vascular endothelial abnormalities are the known molecular mechanisms of endothelial injury in hypertension [1–3]. Increasing evidence suggests that circulating endothelial progenitor cells (EPCs) derived from bone marrow participate in the endothelial repair process in endothelial injury [4–8]. EPCs are able to proliferate and differentiate into endothelial cells and are therefore ideal candidates for application in vascular regeneration [9]. Relevant studies demonstrate that the reendothelialization capability of EPCs is beneficial to maintaining the integrity of the vascular endothelium after arterial injury [8, 9], which is crucial for the prophylaxis and treatment of cardiovascular disease [8, 10–15]. Recent clinical trials proved that the state of hypertension and prehypertension leads to the declined number and dysfunction of circulating EPCs, implying that this impaired endogenous endothelial repair capacity is involved in mediating hypertension-related endothelial dysfunction and vascular injury [4, 16].

Tie2 tyrosine kinase receptor (Tie2) is a significant endothelial-specific receptor tyrosine kinase [9]. Accumulating experimental and clinical evidence supports the hypothesis that Tie2 and its ligands, e.g., angiopoietin-2 (Ang2), contribute to vasculogenesis and angiogenesis [17]. Ang2/Tie2 signalling plays a pivotal role in the biological processes of EPCs, such as chemotactic migration and cell survival [17, 18]. In addition, phosphoinositide 3-kinase (Pl3k), protein kinase B (Akt), and endothelial nitric oxide synthase (eNOS), which are downstream molecules regulating the Ang1-Tie2 signalling pathway, are related to the Ang2-mediated cellular responses of EPCs [9, 10, 17]. Our previous study further demonstrated that the Tie2/PI3k/Akt/eNOS signalling pathway is a target for the shear stress-mediated augmentation of the in vivo reendothelialization capability of transplanted EPCs in endothelial repair [9]. Therefore, the Tie2-dependent pathway plays a crucial role in regulating the endothelial repair capacity of EPCs.

Aliskiren, an active direct renin inhibitor, exhibits beneficial effects on endothelial function, ischaemia-induced neovascularization, and reduced arterial stiffness [19–21]. Aliskiren not only increases the number of EPCs but also improves the function of EPCs in processes such as adhesion and cellular migration [22–24]. However, the mechanism of the beneficial effect of aliskiren upon EPCs is unclear. Therefore, based on previous studies, we hypothesized that aliskiren might improve the endothelial repair capability of human EPCs via the Tie2/PI3k/Akt/eNOS signalling pathway. To test this hypothesis, we focused on aliskiren affecting the in vitro function and in vivo reendothelialization capability of early EPCs from patients with hypertension, evaluated the regulatory effects of aliskiren on the Tie2/Pl3k/Akt/eNOS signalling pathway in EPCs, and studied the role of this signalling pathway in the aliskiren-mediated regulation of EPC function in vitro and reendothelialization capability in vivo in mice. Our present study may thus provide valuable information to the further understanding of cell-based therapy as a novel pharmacological strategy for treating hypertension-related vascular injury.

## 2. Materials and Methods

### 2.1. Characteristics of the Subjects

Eighteen normotensive subjects and eighteen patients with essential hypertension, which had no family history of hypertension, were enrolled. The subjects must have been diagnosed as without cardiovascular diseases or had no ongoing drug and other treatments. The hypertensive patients were diagnosed by sitting blood pressure (after 10 min of rest) measurements obtained three times at 1-week intervals; a systolic blood pressure (SBP) of ≥140 mmHg and (or) a diastolic blood pressure (DBP) of ≥90 mmHg were diagnosed as hypertension. The normotensive subjects had no cardiovascular risk factors, an SBP of <120 mmHg, and a DBP of <80 mmHg, according to the Seventh Report of the Joint National Committee on Prevention, Detection, Evaluation, and Treatment of High Blood Pressure (JNC 7) [25]. The age, sex, and body mass index (BMI) were matched between normotensive subjects and the patients. Complete clinical examination, laboratory tests, and instrumental examination were performed in order to exclude patients with secondary hypertension. Subjects with infection, peripheral artery disease, diabetes, malignant disease, or active inflammatory disorders, as well as those who smoked, were excluded; these conditions above may influence the number or function of EPCs [16]. The consent procedure and experimental protocol were approved by the Ethical Committee of the First Affiliated Hospital of Sun Yat-Sen University (approval no. [2017]078), and written informed consent was collected.

Peripheral venous blood samples from subjects and patients used for EPC isolation and culture as well as for biochemical tests and routine blood, including the measurement of serum total triglyceride, high-density lipoprotein (HDL) cholesterol, low-density lipoprotein (LDL) cholesterol, cholesterol, high-sensitivity C-reactive protein (CRP), and levels fasting plasma glucose (FPG), were obtained from the two groups after overnight fasting [16, 26].

### 2.2. EPC Culture and Identification

EPC isolation and culture were performed as described previously [9, 16, 27–32]. In addition, the procedure for the identification of early EPCs was conducted as described in our previous study [9].

### 2.3. Migration, Proliferation, and Adhesion Assays with Circulating EPCs

The human EPCs cultured for 7 days were pretreated with 1 *μ*mol/L, 10 *μ*mol/L, or 50 *μ*mol/L aliskiren (SIGMA-ALDRICH) for 12 h. EPC migration, proliferation, and adhesion assays were performed as described in previous studies [16, 32–34].

### 2.4. Tie2 Knockdown and Pharmacological Inhibition

The experimental protocols for the knockdown of Tie2 and the inhibition of PI3k and eNOS were performed via the protocol described in our previous study [9]. Tie2 expression was knocked down by shRNA lentiviral transduction particles. The viral transduction (Santa Cruz Biotechnology) was operated according to the manufacturer's manual. The human EPCs were preincubated with the inhibitor for 1 d prior to treatment with different concentrations of aliskiren, namely, 1 *μ*mol/L, 10 *μ*mol/L, and 50 *μ*mol/L. Ten micromolar LY294002 (Calbiochem) was used to inhibit PI3k. eNOS was inhibited by 100 *μ*mol/L L-NAME (Calbiochem).

### 2.5. Western Blot Analysis

As previously described in our studies [9, 26], proteins were extracted from EPCs in a cell lysis solution (Thermo Fisher Scientific). After SDS-PAGE treatment, the protein extract was transferred into PVDF membrane (Cell Signaling Technology). The assay-related antibodies include Tie2 rabbit mAb, phospho-Akt antibody, anti-Akt, phospho-eNOS antibody, and anti-eNOS (1 : 1000) and mouse anti-Tie2 (1 : 1000) (Cell Signaling Technology). HRP-conjugated anti-rabbit IgG (1 : 2000) (Cell Signaling Technology) was incubated to visualize the protein, and the protein was observed by ECL chemiluminescence system (Thermo Fisher Scientific). The intensity of the immunoreactive bands was analysed, and the results for the phospho-Tie2, phospho-Akt, and phospho-eNOS in human EPCs are expressed as the ratio of each phosphorylated protein to that of the corresponding unphosphorylated protein. The statistical comparisons for the Western blot analysis were made relative to non-aliskiren-treated EPCs from patients with hypertension.

### 2.6. Animal Model and Reendothelialization Assay

The nude mouse model of carotid artery injury was generated and the reendothelialization experiment was performed as described in our previous study [9].

All experimental programs were approved by the Animal Care and Use Committee of the First Affiliated Hospital of Sun Yat-Sen University and conformed to the Guide for the Care (approval no. [2017]118) and Use of Laboratory Animals published by the US National Institutes of Health (NIH).

### 2.7. Statistical Analysis

SPSS V20.0 software was used for the statistical analyses. All the values are presented as the means value ± SDs. Student's *t*-test was used to compare the two groups. *P* values of less than 0.05 were considered statistically significant.

## 3. Results

### 3.1. Clinical Characteristics

The baseline characteristics of the two groups indicated that no significant differences were found except for the mean systolic and diastolic pressure. The SBP and DBP measurements were lower in the normotensive group than in the hypertensive group (Table 1).

The repair capacity of EPCs and the phospho-Tie2, phospho-Akt and phospho-eNOS in EPCs were lower in hypertensive patients.

**Figure 1:**
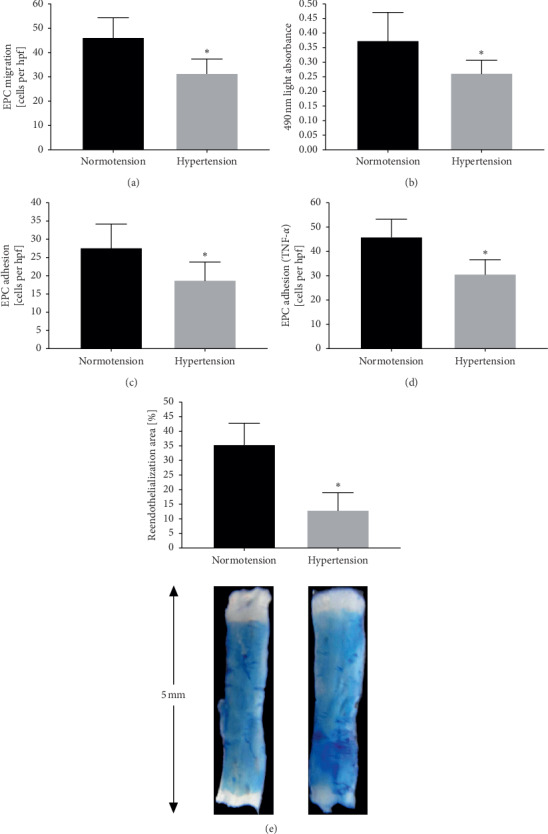
The quantification analysis of the in vitro function and in vivo reendothelialization capacity of human EPCs in the two groups. (a) Quantification analysis of human EPC migration (^*∗*^*P* < 0.05 vs. EPCs from normotensive subjects, *n* = 18 per group). (b) Quantification analysis of the proliferative activity of human EPCs (^*∗*^*P* < 0.05 vs. EPCs from normotensive subjects, *n* = 18 per group). (c)–(d) Quantification analysis of human EPC adhesion to HUVECs with or without TNF-*α* activation (^*∗*^*P* < 0.05 vs. EPCs from normotensive subjects, *n* = 18 per group). (e) Quantification analysis of the area of carotid artery reendothelialization by transplanted EPCs on day 3 after injury (^*∗*^*P* < 0.05 vs. EPCs from normotensive subjects, *n* = 18 per group). hpf = high-power field.

The levels of migration and proliferation of EPCs were lower in the hypertensive than in normotensive (Figures 1(a) and 1(b)). The adhesion activity of EPCs was lower in the hypertensive than in the normotensive. Moreover, after the HUVECs (human umbilical vein endothelial cells) were pretreated with TNF-*α*, the adhesion of EPCs was increased in both the normotensive and hypertensive groups (Figures 1(c) and 1(d)). Compared with EPCs transplanted from patients with hypertension, EPCs transplanted from normotensive subjects had a greater reendothelialization capability, suggesting that hypertension attenuated the reendothelialization capability in vivo of EPCs (Figure 1(e)).

**Figure 2:**
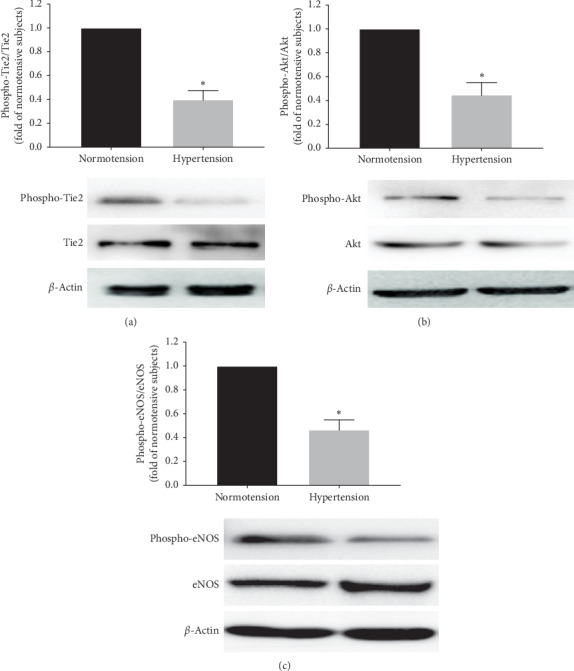
The quantification analysis of Tie2, Akt, and eNOS phosphorylation in human EPCs in the two groups. (a–c) Quantification analysis showing that the phosphorylation of Tie2, Akt, and eNOS in EPCs from patients with hypertension was significantly lower than that in EPCs from normotensive subjects (^*∗*^*P* < 0.05 vs. EPCs from normotensive subjects, *n* = 18 per group).

Similarly, the phospho-Tie2, phospho-Akt, and phospho-eNOS in EPCs were lower in the hypertensive than in the normotensive, respectively (Figures 2(a)–2(c)).

### 3.2. Aliskiren Upregulated the Migration, Proliferation, and Adhesion of EPCs In Vitro

**Figure 3:**
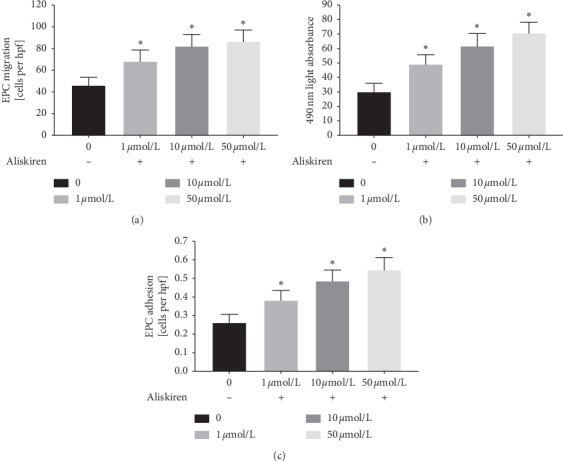
The effect of different concentrations of aliskiren on the in vitro function of patients' EPCs. (a) Quantification analysis of the migration of patients' EPCs treated with 1 *μ*mol/L, 10 *μ*mol/L, or 50 *μ*mol/L aliskiren for 12 h (^*∗*^*P* < 0.05 vs. subjects' EPCs without aliskiren treatment, *n* = 18 per group). Quantification analysis of the proliferation (b) and adhesion (c) of patients' EPCs treated as described above (^*∗*^*P* < 0.05 vs. subjects' EPCs without aliskiren treatment, *n* = 18 per group). hpf = high-power field.

The human EPCs cultured for 7 days were pretreated with 1 *μ*mol/L, 10 *μ*mol/L, or 50 *μ*mol/L aliskiren for 12 h. Treatment with aliskiren resulted in a dose-dependent increase in the migratory (Figure 3(a)) and proliferative (Figure 3(b)) activity of EPCs, respectively. Furthermore, aliskiren markedly enhanced the adhesion of EPCs (Figure 3(c)).

### 3.3. Aliskiren Increased the Phospho-Tie2, Phospho-PI3k, and Phospho-Akt in EPCs

**Figure 4:**
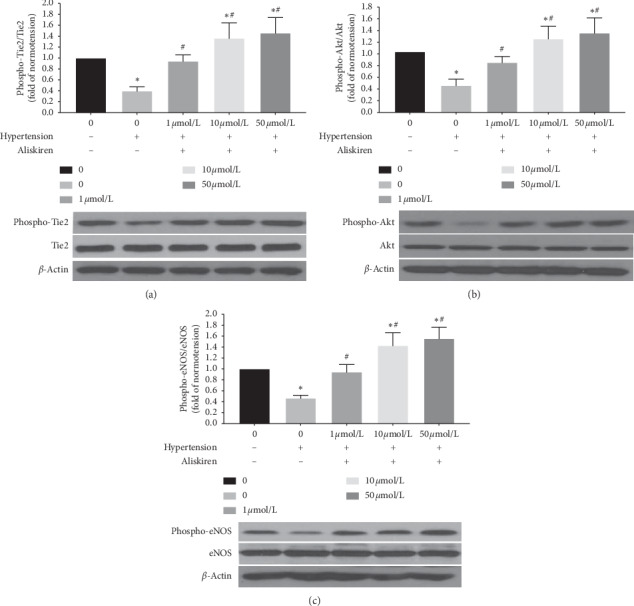
The quantification analysis of Tie2, Akt, and eNOS phosphorylation in patients' EPCs treated with different concentrations of aliskiren. Quantification analysis of Tie2 (a), Akt (b), and eNOS (c) phosphorylation in patients' EPCs treated with 1 *μ*mol/L, 10 *μ*mol/L, or 50 *μ*mol/L aliskiren for 12 h (^*∗*^*P* < 0.05 vs. non-aliskiren-treated EPCs from normotensive subjects, *n* = 18; ^#^*P* < 0.05 vs. non-aliskiren-treated EPCs from hypertensive patients, *n* = 18).

Aliskiren has a favourable effect on the phospho-Tie2, phospho-Akt, and phospho-eNOS in EPCs from patients with hypertension. The phospho-Tie2, phospho-Akt, and phospho-eNOS in EPCs were markedly lower in the hypertensive than in the normotensive (Figures 4(a)–4(c)). When EPCs from patients with hypertension were treated in vitro with 1 *μ*mol/L, 10 *μ*mol/L, or 50 *μ*mol/L aliskiren for 12 hours, the phospho-Tie2, phospho-Akt, and phospho-eNOS were enhanced in a dose-dependent manner. For concentrations of 10 *μ*mol/L or 50 *μ*mol/L aliskiren, the phospho-Tie2, phospho-Akt, and phospho-eNOS in EPCs from hypertensive patients were markedly higher than those in EPCs from normotensive subjects, and the level of phosphorylation was relatively high for aliskiren concentrations of both 10 *μ*mol/L and 50 *μ*mol/L (Figures 4(a)–4(c)).

### 3.4. Aliskiren Modulated the PI3k/Akt/eNOS Signalling Pathway in EPCs

**Figure 5:**
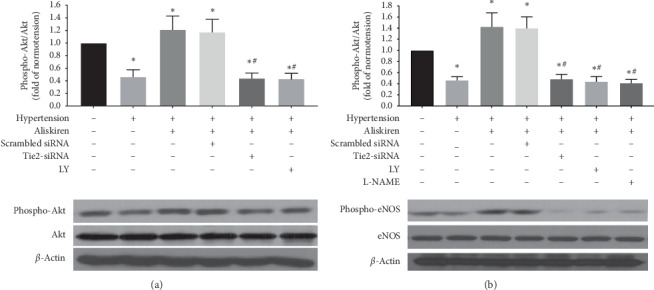
The role of the Tie2/PI3k/AKT pathway in the aliskiren-induced phosphorylation of eNOS. (a) Quantification analysis of aliskiren-induced Akt phosphorylation in EPCs with Tie2 knockdown or after PI3k inhibition (LY294002) (^*∗*^*P* < 0.05 vs. non-aliskiren-treated EPCs from normotensive subjects, *n* = 18 per group;^#^*P* < 0.05 vs. aliskiren-treated EPCs from hypertensive patients, *n* = 18 per group). (b) Quantification analysis of aliskiren-induced eNOS phosphorylation in EPCs with Tie2 knockdown or after PI3k or eNOS inhibition (LY-NAME) (^*∗*^*P* < 0.05 vs. non-aliskiren-treated EPCs from normotensive subjects, *n* = 18 per group; ^#^*P* < 0.05 vs. aliskiren-treated EPCs from hypertensive patients, *n* = 18 per group).

We further investigated whether the aliskiren-induced phosphorylation of eNOS was regulated by the Tie2/PI3k/Akt pathway via Tie2 knockdown and PI3k and eNOS inhibition experiments. Treatment with aliskiren for 12 h increased the phospho-Akt and phospho-eNOS from patients with hypertension (Figures 5(a) and 5(b)). No significant differences were found in the phospho-Akt and phospho-eNOS treated with or without scrambled siRNA (Figures 5(a) and 5(b)). The increased phospho-Akt and phospho-eNOS induced by aliskiren were reduced by 63% and 64%, respectively, after Tie2 knockdown (Figures 5(a) and 5(b)), suggesting that Tie2 knockdown abolished the aforementioned upregulation of Akt and eNOS phosphorylation. Furthermore, PI3k inhibition suppressed the favourable effect of aliskiren on the phospho-Akt and phospho-eNOS (Figures 5(a) and 5(b)). The increased phospho-Akt and phospho-eNOS treated with aliskiren were reduced by 64% and 67%, respectively, after treatment with LY294002. Likewise, the increase in phospho-eNOS treated with aliskiren was suppressed by L-NAME (Figure 5(b)). The above results indicated that aliskiren upregulates the phospho-Akt and phospho-eNOS in EPCs from patients with hypertension via the Tie2/PI3k/Akt/eNOS signalling pathway.

### 3.5. The Tie2/PI3K/Akt/eNOS Signalling Pathway Contributed to the Aliskiren-Induced Upregulation of the In Vitro Function and In Vivo Reendothelialization Capability of Human EPCs

**Figure 6:**
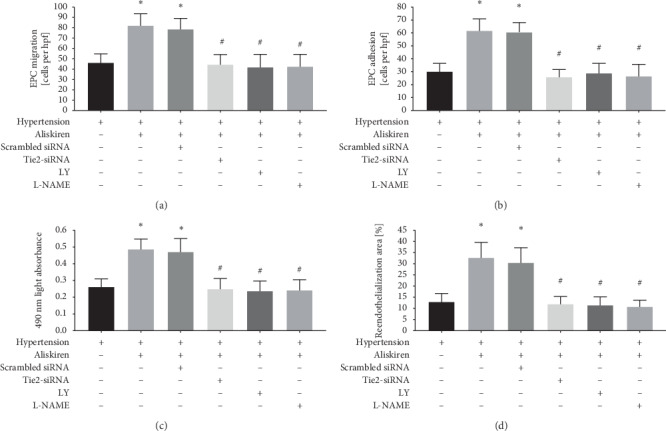
Tie2/PI3k/AKT signalling pathway blockade inhibits the in vitro function and in vivo reendothelialization capacity of EPCs treated with aliskiren. (a–c) Quantification analysis of aliskiren-mediated migration (^*∗*^*P* < 0.05 vs. non-aliskiren-treated EPCs from normotensive subjects, *n* = 18 per group; ^#^*P* < 0.05 vs. aliskiren-treated EPCs from hypertensive patients, *n* = 18 per group) (a), adhesion (^*∗*^*P* < 0.05 vs. non-aliskiren-treated EPCs from normotensive subjects, *n* = 18 per group; ^#^*P* < 0.05 vs. aliskiren-treated EPCs from hypertensive patients, *n* = 18 per group) (b), and proliferation (^*∗*^*P* < 0.05 vs. non-aliskiren-treated EPCs from normotensive subjects, *n* = 18 per group; ^#^*P* < 0.05 vs. aliskiren-treated EPCs from hypertensive patients, *n* = 18 per group) (c) of EPCs with Tie2 knockdown or after PI3k or eNOS inhibition. hpf = high-power field. (d) Quantification analysis of the aliskiren-mediated reendothelialization capacity of EPCs with Tie2 knockdown or after PI3k or eNOS inhibition (^*∗*^*P* < 0.05 vs. non-aliskiren-treated EPCs from normotensive subjects, *n* = 18 per group; ^#^*P* < 0.05 vs. aliskiren-treated EPCs from hypertensive patients, *n* = 18 per group).

We hypothesized that aliskiren upregulated this signalling pathway to enhance the in vitro function and in vivo reendothelialization capability of human EPCs. The results indicated that aliskiren exerted significant beneficial effects on the in vitro migratory, adhesive, and proliferative activity as well as on the in vivo reendothelialization capability of EPCs (Figures 6(a)–6(d)). However, Tie2 knockdown and PI3k inhibition (LY294002) or eNOS inhibition (L-NAME) suppressed the increase in all the in vitro functions of aliskiren-treated EPCs (Figures 6(a)–6(c)). Similarly, they eliminated the increase in the in vivo reendothelialization capability of transplanted EPCs treated with aliskiren (Figure 6(d)). The transduction of the scrambled siRNA control for the Tie2-siRNA lentiviral particles did not have a statistically significant effect (Figures 6(a)–6(d)).

## 4. Discussion

Our findings indicated that aliskiren could enhance the in vitro migratory, proliferative, and adhesive activity of EPCs from patients with hypertension and increase the in vivo reendothelialization area of human EPCs in a nude mouse model. Similarly, the phospho-Tie2, phospho-AKt, and phospho-eNOS in EPCs were increased as the result of aliskiren. After blockade of the Tie2/PI3k/Akt/eNOS signalling pathway, the favourable effect of aliskiren on the in vitro function and in vivo reendothelialization capability of EPCs was consequently inhibited. Thus, aliskiren can improve the in vitro function and in vivo reendothelialization capability of EPCs from patients with hypertension at least partly through the Tie2/PI3k/Akt/eNOS signalling pathway.

This study indicated that the number and function of EPCs was reduced in patients with essential hypertension [4], which further confirmed the diminished endogenous endothelial repair capacity in hypertension [16]. In addition, our results revealed that the phospho-Tie2, phospho-Akt, and phospho-eNOS in EPCs were reduced in patients with hypertension; this reduction might be related to the impaired function and endothelial repair capacity of EPCs. Therefore, the downregulation of the Tie2/PI3K/AKt/eNOS signalling pathway in EPCs is the crucial mechanism underlying the reduced endogenous vascular repair capacity in hypertension.

Previous studies demonstrated that aliskiren has a favourable effect on endothelial function in patients with essential hypertension by improving the carotid-femoral pulse wave velocity (cfPWV), the reactive hyperaemia peripheral arterial tonometry (RH PAT) index, and flow-mediated dilatation (FMD). Aliskiren has been proven to enhance the number of EPCs [19–21], which may be responsible for its favourable effect on endothelial function. However, the underlying mechanism of the beneficial effect of aliskiren is still unclear. Tie2-dependent signalling has been generally accepted to contribute to the increase in EPC function and subsequent in vivo angiogenesis [9, 17, 1 8, 35–37]. Moreover, recent studies indicated that aliskiren could activate the PI3K/Akt/eNOS signalling pathway in spontaneously hypertensive rats (SHRs) [38]. Accordingly, we hypothesized that Tie2, one of the upstream signalling mediators of the PI3K/Akt/eNOS pathway, might be activated by aliskiren and might subsequently regulate the PI3K/Akt/eNOS pathway, thus leading to an increase in the in vitro function and in vivo reendothelialization capability of EPCs in patients with hypertension. To address this hypothesis, the effect of aliskiren on the Tie2/PI3K/Akt/eNOS pathway in EPCs from patients with hypertension was investigated. Similar to the results of previous studies, we found that aliskiren can enhance the in vitro migratory activity, proliferative activity, and adhesion of EPCs in a dose-dependent manner in patients with hypertension. Furthermore, aliskiren can accelerate the reendothelialization mediated by EPCs in patients with hypertension, indicating that the aliskiren-induced increased endothelial repair capacity was associated with its beneficial effect on the in vitro function of EPCs. Additionally, aliskiren can increase the phospho-Tie2, phospho-Akt, and phospho-eNOS in EPCs in a dose-dependent manner, which was in parallel with its effect on the in vitro function. Accordingly, the aliskiren-mediated increase in EPC function was related to alterations in the Tie2/PI3K/Akt/eNOS signalling pathway. In order to further verify this hypothesis, we first investigated the modulatory effect of aliskiren on Tie2-dependent signalling. When blocked by Tie2-siRNA or inhibited by LY, the aliskiren-mediated increase in phospho-Akt and phospho-eNOS was abolished, suggesting that aliskiren activates the phospho-Akt and phospho-eNOS via Tie2-dependent signalling. These results confirmed the regulatory effect of aliskiren on the Tie2/PI3K/Akt/eNOS pathway in EPCs from patients with hypertension.

Furthermore, when this signalling pathway was blocked, the aliskiren-induced enhancement in the in vitro migratory, proliferative, and adhesive activity of EPCs from patients with hypertension was inhibited, indicating the role of Tie2-dependent signalling in aliskiren-regulated EPC function in the setting of hypertension. Similarly, the increased EPC-mediated reendothelialization in patients with hypertension was attenuated after the blockade of this signalling pathway, further supporting the relationship between Tie2-dependent signalling and the endothelial repair capacity. The present results suggested that the beneficial effect of aliskiren on increasing both the in vitro function and the in vivo reendothelialization capability of EPCs are at least partly regulated by the Tie2/PI3K/Akt/eNOS signalling pathway.

The findings obtained in this study provide some valuable information, as follows. First, our study revealed that hypertension leads the diminished phosphorylation level of Tie2, Akt, and eNOS in EPCs and then leads to a reduction in the in vitro function and in vivo reendothelialization capability of EPCs; this process is the crucial mechanism underlying the reduced vascular repair capacity in the hypertensive. Second, aliskiren can activate the Tie2 signalling pathway and subsequently increase the in vitro function as well as the in vivo reendothelialization capability of EPCs from patients with hypertension, which indicates the favourable effect of aliskiren on the endothelial repair capacity in the setting of hypertension, as well as its possible mechanism. The present findings suggest an important pharmacological therapeutic target for the EPC-based repair for hypertension-related vascular damage.

However, this study has some limitations. First, because of the restriction on the use of aliskiren in clinical trials in China, our study did not test the effect of the oral administration of aliskiren on the Tie2/PI3K/Akt/eNOS pathway in circulating EPCs in patients with hypertension. However, the accelerated reendothelialization mediated by circulating EPCs, along with the relationship between this increase in reendothelialization capability and the Tie2/PI3K/Akt/eNOS signalling pathway in response to in vitro treatment with aliskiren, can partly elucidate both the favourable effect of aliskiren on the endothelial repair capability and its underlying mechanism. Second, early EPCs play important roles in endothelial repair processes [39], and late-outgrowth EPCs are involved in angiogenesis in response to the stimulus of ischaemia. In this investigation, we did not study the effect of aliskiren on the number and function of late-outgrowth EPCs. A recent study demonstrated that aliskiren can not only increase the number of EPCs but can also improve ischaemia-induced neovascularization in mice with diabetes via an SDF-1-related mechanism [22]; however, the populations of EPCs used in this study were not clearly stated. In addition, the Tie2 signalling pathway has been reported to contribute to vasculogenesis and angiogenesis [17]. Accordingly, aliskiren can be inferred to increase the number and function of late-outgrowth EPCs and subsequently promote ischaemia-induced neovascularization via the Tie2 signalling pathway. The effect of aliskiren on late-outgrowth EPCs, as well as the underlying mechanism, remains to be further investigated.

## 5. Conclusion

This study, for the first time, indicates that aliskiren can improve the in vitro function and in vivo reendothelialization capability of circulating EPCs in patients with hypertension via the Tie2/PI3k/Akt/eNOS signalling pathway. All our findings indicate that Tie2-dependent signalling is a crucial target for the EPC-based repair of hypertension-related endothelial injury, as well as provide new insights into a pharmacological therapeutic approach for treating vascular injury in hypertension.

## Figures and Tables

**Table 1:** Clinical and biochemical characteristics.

Characteristics	Normotensive subjects (*n* = 18)	Hypertensive patients (*n* = 18)
Age (years)	54.9 ± 7.9	55.3 ± 8.1
Height (cm)	166.8 ± 6.4	163.8 ± 6.0
Weight (kg)	63.1 ± 6.6	63.2 ± 5.5
BMI (kg/cm^2^)	22.7 ± 2.4	23.6 ± 2.3
Systolic blood pressure (mmHg)	120.4 ± 11.1	148.6 ± 6.3^*∗*^
Diastolic blood pressure (mmHg)	77.3 ± 6.8	90.4 ± 4.9^*∗*^
Heart rate (beats/min)	71.3 ± 9.0	72.9 ± 7.8
AST (mmol/L)	25.3 ± 5.8	25.8 ± 5.8
ALT (mmol/L)	22.4 ± 4.1	23.5 ± 4.8
BUN (mmol/L)	5.34 ± 1.14	5.66 ± 1.16
Cr (mmol/L)	59.4 ± 12.8	62.4 ± 12.0
LDL (mmol/L)	2.89 ± 0.46	3.05 ± 0.40
TC (mmol/L)	4.84 ± 0.59	5.00 ± 0.48
HDL (mmol/L)	1.46 ± 0.22	1.42 ± 0.21
TG (mmol/L)	1.40 ± 0.18	1.44 ± 0.17
FPG (mmol/L)	4.95 ± 0.67	4.73 ± 0.73

Abbreviation: BMI, body mass index; LDL, low-density lipoprotein; TC, total cholesterol; HDL, high-density lipoprotein; TG, triglyceride; FPG, fasting plasma glucose. Notes: data are given as mean ± SD. ^*∗*^vs normotensive subjects.

## Data Availability

The datasets used and/or analyzed during the current study are available from the corresponding author upon reasonable request.

## Abbreviations

Ang2: Angiopoietin-2
Akt/PKB: Protein kinase B
EPCs: Endothelial progenitor cells
eNOS: endothelial nitric oxide synthase
HUVECs: Human umbilical vein endothelial cells
PI3K: Phosphatidylinositol 3′-kinase
Tie2: Tyrosine kinase with immunoglobulin and epidermal growth factor homology domain-2.
